# Pathological Complete Response and Long-Term Survival in a Very Elderly Patient after Neoadjuvant Chemotherapy for Locally Advanced, Unresectable Gastric Cancer

**DOI:** 10.1155/2014/532924

**Published:** 2014-09-14

**Authors:** Kunihiko Izuishi, Mitsuyoshi Kobayashi, Takanori Sano, Hirohito Mori, Kazuo Ebara

**Affiliations:** ^1^Department of Gastroenterological Surgery, Federation of Public Services and Affiliated Personnel Aid Associations, Takamatsu Hospital, 4-18 Tenjinmae, Takamatsu, Kagawa 760-0018, Japan; ^2^Department of Internal Medicine of Gastroenterology, Federation of Public Services and Affiliated Personnel Aid Associations, Takamatsu Hospital, 4-18 Tenjinmae, Takamatsu, Kagawa 760-0018, Japan

## Abstract

We address the pathological complete response and long-term survival of elderly patients after neoadjuvant chemotherapy in locally advanced, unresectable gastric cancer. An 83-year-old man was hospitalized for upper abdominal pain. Gastrointestinal endoscopy showed a large tumor spanning from the gastric angle to the antrum, and extending to the duodenum. Histological analysis of the biopsy specimen revealed a poorly differentiated adenocarcinoma. Computed tomography images showed thickening of the gastric wall and invasion of the body and head of the pancreas, but did not show distant metastases. The patient was diagnosed with unresectable gastric cancer, and was treated with neoadjuvant chemotherapy using S-1 (80 mg/m^2^) and paclitaxel (60 mg/m^2^). After the third course of chemotherapy, gastrointestinal endoscopy and abdominal computed tomography revealed a remarkable reduction in tumor size. This reduction allowed distal gastrectomy to be conducted. Histological examination of the specimen revealed no cancer cells in the primary lesion or lymph nodes. The patient was treated with adjuvant chemotherapy of oral tegafur-uracil (300 mg/day) for one year after surgery. He lived for five years after surgery without recurrence. Neoadjuvant chemotherapy using S-1 and paclitaxel is a potent strategy for improving survival in very elderly patients with unresectable gastric cancer.

## 1. Introduction

Increased life expectancy has led to many opportunities for treating gastric cancer in very elderly patients (≥80 years old). This report details the case of an 83-year-old male patient who showed a pathological complete response after neoadjuvant chemotherapy (NAC) and achieved long-term survival after locally advanced, unresectable gastric cancer.

Gastric cancer generally has a poor prognosis [1, 2]. Many patients with gastric cancer are inoperable at initial diagnosis due to remote metastases and/or direct invasion of adjacent organs. However, the development of new chemotherapeutic agents has significantly prolonged the survival of patients with far-advanced gastric cancer. The combination therapy of S-1 (a combination of three pharmacological compounds: tegafur, gimeracil, and oteracil potassium) plus cisplatin (CDDP) showed significant improvements in the prognoses of inoperable advanced gastric cancer patients [3]. The combination therapy of trastuzumab (a monoclonal antibody against human epidermal growth factor receptor 2 (HER2)) and chemotherapy prolonged the survival of patients with HER2-positive advanced gastric cancer [4]. These results open new fields of treatment strategies for patients with far-advanced cancer. However, long-term survival is difficult for these patients. Resection is the only curative strategy for gastric cancer. Therefore, for locally advanced patients without remote metastases, preoperative downstaging via NAC is a potent strategy for performing curative surgery [2]. However, pathological complete response is very rare [5–7], and five-year survival without recurrence has not been reported. It is possible that some cancer cells remain in patients after surgery, allowing for eventual recurrence. This report will discuss strategies for treating gastric cancer in very elderly patients, including details of this rare case.

## 2. Case Report

**Figure 1:**
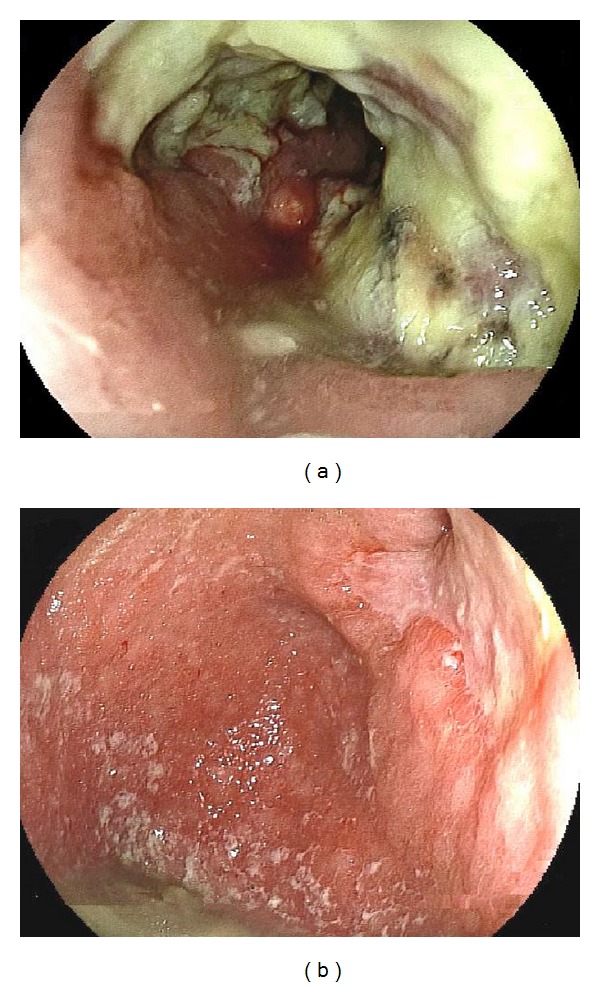
Upper gastrointestinal endoscopy. (a) Huge ulcerated tumor from the gastric angle to the antrum, extending to the duodenum, was seen before chemotherapy. (b) A remarkable reduction of tumor size was seen after the third course of chemotherapy.

**Figure 2:**
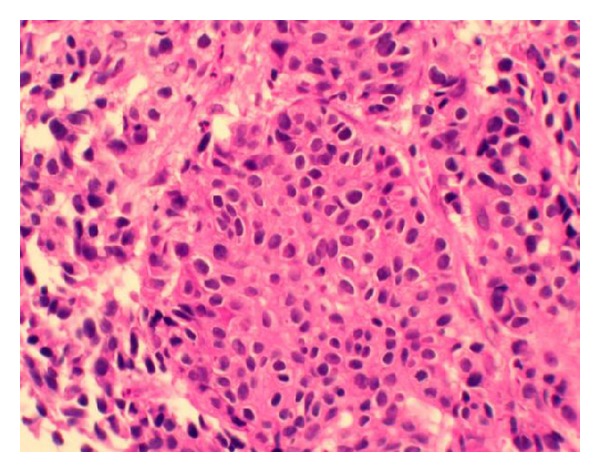
Histological analysis of the biopsy specimen. Microscopic examination showed a poorly differentiated adenocarcinoma.

**Figure 3:**
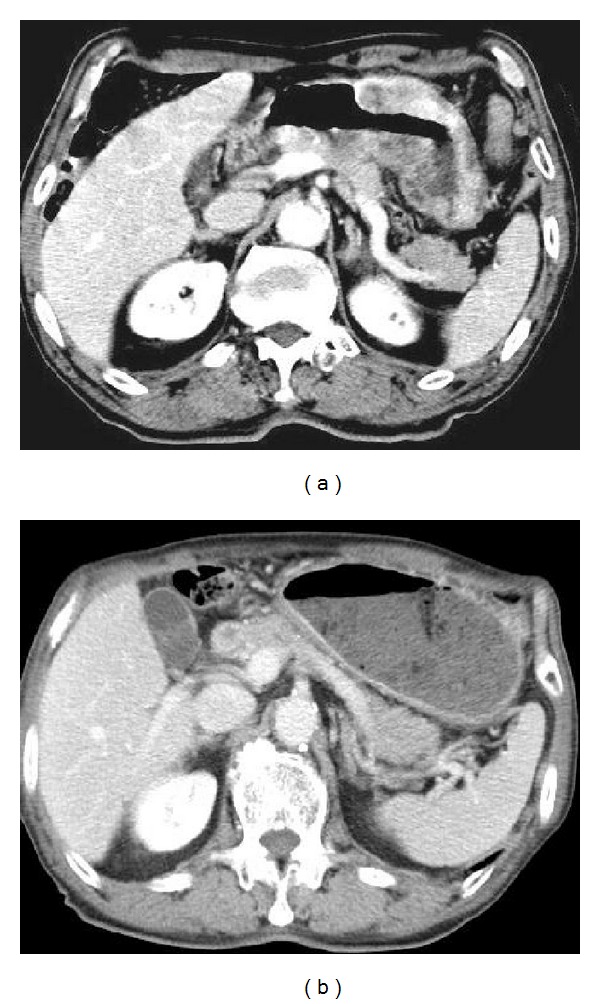
Computed tomography (CT) imaging. (a) The remarkable thickened gastric wall which suggested the invasion of the pancreas was depicted before chemotherapy. (b) Normalized gastric wall and separated gastric wall and pancreas was seen after chemotherapy.

**Figure 4:**
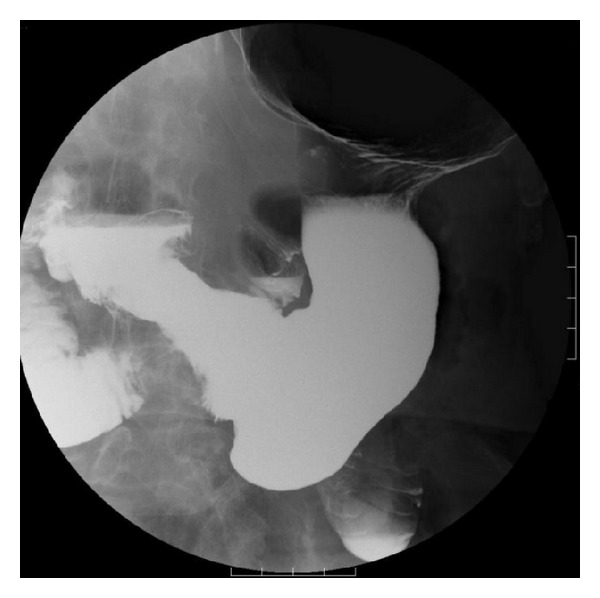
Fluoroscopy of the upper gastrointestinal tract. The huge tumor, 10 cm in diameter, was located in the antrum of stomach.

An 83-year-old man was hospitalized for upper abdominal pain and weight loss of 4 kg over three months. His past history included pulmonary emphysema. A physical examination revealed a hard, palpable mass in the middle upper abdomen of approximately 10 cm in diameter. Laboratory data showed an elevated leukocyte count of 10,800 per mm^3^, a decreased hemoglobin level of 11.8 g/dL, and a normal tumor marker level of carcinoembryonic antigen and carbohydrate antigen 19-9. Upper gastrointestinal endoscopy showed a large, ulcerated, tumor spanning from the gastric angle to the antrum and extending to the duodenum (Figure 1(a)). Histological analysis of the biopsy specimen revealed a poorly differentiated adenocarcinoma (Figure 2). Computed tomography (CT) imaging showed a thickened gastric wall, invasion of the pancreas, and lymph nodes swelling at greater curvature of the stomach but did not show any distant metastases (Figure 3(a)). Fluoroscopy of the upper gastrointestinal tract allowed visualization of the tumor, which was located in the stomach and was 10 cm in diameter (Figure 4). This imaging also showed adhesion of the stomach and pancreas. Abdominal ultrasonography also suggested invasion of the pancreas. Based on the above findings, the mass was determined to have invaded the head and body of the pancreas, and the clinical stage was T4bN1M0, stage IIIB (Union for International Cancer Control (UICC)). The patient was diagnosed with unresectable gastric cancer, and chemotherapy with S-1 and paclitaxel was initiated. Oral S-1 (80 mg/m^2^) was administered for two weeks, and intravenous (IV) paclitaxel (60 mg/m^2^) was administered on days one and eight. The patient received three cycles of this regimen at 14-day intervals and showed side effect of fatigue (grade 2 based on common terminology criteria for adverse events). Assessment of the patient's response to chemotherapy after the third course of treatment showed a remarkable reduction in tumor size by gastrointestinal endoscopy (Figure 1(b)). Abdominal CT showed decreased thickness of the gastric wall and a more distinct border between the pancreas and stomach (Figure 3(b)). Based on these findings, laparotomy was conducted. A weak adhesion was found between the stomach and pancreas during the procedure, but curative surgery was possible. Following the laparotomy, a distal gastrectomy was conducted. The patient's postoperative course was uneventful and he was discharged 20 days after surgery. The patient received adjuvant chemotherapy of oral tegafur-uracil (300 mg/day) for one year without any side effects. He lived for five years after surgery without recurrence.

### 2.1. Histopathological Findings

**Figure 5:**
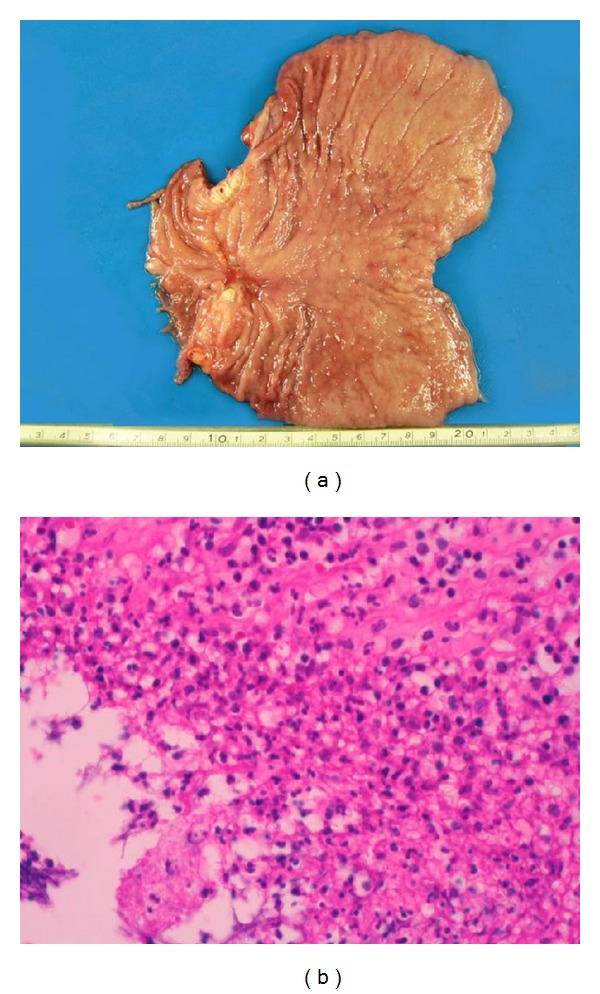
The appearance resected specimen. (a) The gross appearance showed an ulcerative lesion 3 cm in diameter at the antrum of stomach. (b) Histological examination of the specimen revealed no cancer cells in the primary lesion or lymph nodes. Marked lymphocytic infiltration and fibrosis were seen in the ulcerative lesion.

The gross appearance of the resected specimen showed an ulcerative lesion 3 cm in diameter at the antrum of stomach (Figure 5(a)). Histological examination of the specimen revealed no cancer cells in the primary lesion or lymph nodes (Figure 5(b)). Fibrosis with marked lymphocytic infiltration was seen in the ulcerative lesion.

## 3. Discussion

Preoperative chemotherapy has been studied and performed in many hospitals [2, 5–7]. Previous reports of NAC have included combinations of chemotherapeutic agents such as S-1, cisplatin, methotrexate, fluorouracil, paclitaxel, epirubicin, and leucovorin [8]. The Medical Research Council Adjuvant Gastric Infusional Chemotherapy trial, which investigated the efficacy of perioperative chemotherapy for operable gastric cancer and lower esophageal cancer, used a combination of epirubicin, cisplatin, and fluorouracil [9]. The perioperative chemotherapy was shown to decrease tumor size and stage and improve progression-free survival and overall survival. The European Organization for Research and Treatment of Cancer Randomized Trial 40954, which used a combination of cisplatin, folinic acid, and fluorouracil, showed a significantly increased R0 resection rate but failed to show an improved overall survival rate [10]. Therefore, the benefits of NAC have yet to be defined in a clinical study. In Japan, S-1 is a key drug for the treatment of advanced gastric cancer. Because, single-agent S-1 is an effective adjuvant treatment for patients after gastrectomy [11], notably, treatment using a combination of S-1 and CDDP has yielded positive results in more than 50% of patients with unresectable gastric cancer [3]. Therefore, this protocol is mainly used for NAC in Japan.

In the current case, paclitaxel was combined with S-1 based on previous protocol [12]. The reason for this choice was the patient's age of 83 years. To avoid acute renal failure, the patient needed to maintain hydration by ingesting over 3000 mL/day during CDDP chemotherapy. The risk of overhydration is high in very elderly people, because of age-related decreases in cardiac function. Frequent urination caused by increased hydration may also cause discomfort in very elderly people due to prostatomegaly. Additionally, monitoring of age-related decreases in renal function is critical during administration of CDDP. In the current case, the patient's creatinine level was slightly elevated (0.9–1.1), and reduced kidney size was revealed by CT examination.

S-1 consists of three drugs: tegafur (a prodrug of fluorouracil), gimeracil (which inhibits fluorouracil degradation), and oteracil (which reduces gastrointestinal toxicity caused by fluorouracil phosphorylation) [13]. Once renal function deteriorates due to CDDP, the concentration of fluorouracil increases, leading to increased fluorouracil toxicity (because gimeracil is excreted in urine via the kidney) [13]. As a result, it may be necessary to avoid the use of S-1, the key medication, in certain patients. Paclitaxel is metabolized by cytochrome P450 (CYP) in the liver [14]; thus chemotherapy using paclitaxel requires less hydration, and renal failure occurs less frequently than with CDDP use. In the current case, paclitaxel was selected for NAC. It is possible that NAC using S-1 and paclitaxel may be a potent strategy for treating very elderly patients with gastric cancer. Randomized trials with large numbers of participants will be needed to determine the efficacy of NAC in patients with locally advanced gastric cancer.

The rate of aging population is increasing in many countries due to the progress of medicine and public health. In addition, aging is the risk factor for cancer. Therefore, it is important to conduct the effective and well-tolerable chemotherapy for elderly patients. Elderly patients do not have tolerance for chemotherapy compared with younger patients, because of the reduced organ function, such as heart, liver, kidney, and bone-marrow, related to age and comorbidities. Therefore, the delicate balance between survival benefit and toxicity is very important in chemotherapy for elderly patients. Usually, the patient over 80 years has been excluded from clinical trial. The optimal chemotherapy for elderly gastric cancer patients is not still elucidated. Therefore, the further clinical study will be needed urgently to establish the secure chemotherapy for elderly gastric cancer patients before the advent of an aging society.

In conclusion, the optimum treatment for locally advanced gastric cancer is still debated. Extended gastric resection with other organs cannot be performed in very elderly patients. NAC has shown promise as a strategy for treating advanced gastric cancer. This report details a case of pathological complete response and long-term survival after NAC using S-1 plus paclitaxel in a case of unresectable, locally advanced gastric cancer. In addition, this combination therapy was performed safely in an elderly patient aged >80 years.
